# Habitat, Snow-Cover and Soil pH, Affect the Distribution and Diversity of Mortierellaceae Species and Their Associations to Bacteria

**DOI:** 10.3389/fmicb.2021.669784

**Published:** 2021-07-01

**Authors:** Anusha Telagathoti, Maraike Probst, Ursula Peintner

**Affiliations:** Institute of Microbiology, University of Innsbruck, Innsbruck, Austria

**Keywords:** Mortierellomycotina, bacterial interactions, winter, pH, altitude, alpine, sub-alpine, psychrotolerant

## Abstract

Mortierellaceae species are among the most frequent and globally distributed soil fungi. However, the factors shaping their diversity and distribution remain obscure. Several species have been reported to be associated to bacteria, but the kind and frequency of such associations were not addressed up to now. We hypothesized that such associations could be important for Mortierellaceae ecology. Therefore, our aim was to understand the driving factors responsible for the Mortierellaceae diversity, community composition and bacterial associations in alpine and subalpine habitats. For answering our question, we collected both snow-free and snow-covered soil at sampling sites from different habitats: bare alpine soil in a glacier forefield, alpine dwarf-willow habitats, and high-altitude *Pinus cembra* forests. The isolations were carried out by direct cultivation without any antibiotics to the isolation media. Altogether, we obtained 389 Mortierellaceae isolates representing 29 operational taxonomic units (OTUs). Many OTUs could be placed to the genera *Mortierella sensu stricto*, *Dissophora, Entomortierella, Gamsiella, Linnemannia*, and *Podila*, but others could not unambiguously be assigned to a genus. Our results demonstrate that both, the distribution as well as the diversity of the Mortierellaceae species, were significantly influenced by habitat, soil pH, and snow-cover. We noticed that >30% of our isolates were associated to a non-contaminant bacterium. The bacteria associated to our Mortierellaceae isolates belonged to seven different genera. *Pseudomonas* was the most frequently detected genus associated to the isolated Mortierellaceae species and it was found to be species-specific. Mortierellaceae–bacteria pairs, including those with *Pseudomonas*, were influenced by location, habitat, and snow-cover. The majority of the fungus–bacterium associations were potentially epihyphal, but we also detected potential endohyphal bacterial species belonging to *Mycoavidus, Burkholderiaceae*, and *Paraburkholderia*. Taken together, the non-random associations we detected suggest that fungus–bacterium associations are ecologically meaningful – an interesting path that needs to be investigated further.

## Introduction

Mortierellaceae is a widespread and frequent fungal genus belonging to Mortierellomycotina. It has a global distribution and is a member of the core fungal soil community (Tedersoo et al., 2014). It is a well-studied genus, with a solid database allowing for reliable species identification. Most of the type strains have been sequenced and reference sequences are available in UNITE or other sequence databases. However, the most comprehensive molecular phylogenetic analysis for this group (Wagner et al., 2013) revealed that only a part of the Mortierellaceae diversity is currently known.

A recent study showed that, based on a phylogenomic approach, the genus *Mortierella* is polyphyletic, justifying its splitting and the description of several new genera (Vandepol et al., 2020). We have used this revised taxonomy throughout the manuscript where possible. However, the correct generic placement of many *Mortierella* species is still obscure. In this study, this is the case for *M. angusta, M. bainieri, M. gemmifera, M. pseudozygospora, M. solitaria* and *M. zonata.* For the sake of clarity and consistency, we refer to these species as *Mortierella sensu lato* (*s.l.*) (*Mortierella* in the classical sense). *Mortierella*, as defined in a modern taxonomic concept (Vandepol et al., 2020) is addressed as *Mortierella sensu stricto* (*s.s.*).

Mortierellaceae are very often detected in mycobiome sequencing data obtained from different environments. However, their diversity can often not be resolved, as mycobiome studies are usually based on a short region of the ITS region, in which members of the Mortierellaceae are highly similar. As cultivation data are also scarce (Hibbett and Glotzer, 2011), the ecology of this interesting group of fungi is not well understood. To overcome the problem of lacking type collections, Hibbett and Glotzer (2011) proposed sequence-based classification. Sequence-based classification would be fast and high-throughput, compared to classification based on a physical specimen. However, this methodology has the disadvantage of lacking information on the morphology and function. Given that Mortierellaceae are usually easily cultivable in the lab, studies based on isolates can contribute tremendously to understanding their ecology, because the thread of cultivation bias is lower compared to other less-easily cultivable organisms, although it might not be negligible. In addition, physical specimens, especially living fungal isolates, allow thorough studies on the physiology, metabolite production, or interactions of organisms representing a species concept. This can open interesting and new perspectives concerning the ecological function and interactions of these fungi.

Mortierellaceae, as an example, are classically regarded as saprobial soil fungi. As saprotrophs in the soil, Mortierellaceae drive soil carbon cycling, P-dissolution and immobilization, lipid metabolism, and chitin degradation (Uehling et al., 2017; Zhang et al., 2020). However, Mortierellaceae have also been reported to occur in the rhizosphere of plants (Kwaśna, 2001; Summerbell, 2005a), and in close association to ectomycorrhizae (Ohara, 1967; Summerbell, 2005b). Similar to arbuscular mycorrhizal fungi, Mortierellaceae spp. support phosphate uptake into plants, which may contribute to increased biomass production (Zhang et al., 2011; Johnson et al., 2019). The exact mechanisms of how these beneficial effects are triggered are still unclear, but a dual lifestyle of selected Mortierellaceae spp. as plant-endophytes and saprotrophs could constitute an important trait (Zhang et al., 2020).

On the other hand, Mortierellaceae spp. are not only interacting with other fungi and plants, but they are also tightly interacting with different species of bacteria (Bonfante and Desiro, 2017). Very little information in this regard is currently available, but a recent study (surveying 238 isolates) proved that 20 species of Mortierellaceae isolated from Japanese soil ecosystems harbor Burkholderiaceae-related endobacteria (Takashima et al., 2018). A comparative genomic analysis suggested an ancient origin of the association between *Linnemannia elongata* and its endosymbiont *Mycoavidus cysteinexigens* likely underpinning their close interaction (Uehling et al., 2017).

Synergistic or mutualistic associations with bacteria appear to be comparatively common in Mucoromycotina and Glomeromycotina (Bonfante and Desiro, 2017). *Bifiguratus*, a recently discovered monotypic fungal genus of Mucoromycotina, is also characterized by a dual, endophytic, and soil-dwelling lifestyle. This fungus is associated with a wide variety of endo- and epihyphal bacteria (Torres-Cruz et al., 2017). This publication adds further evidence to the hypothesis, that fungus–bacteria associations are common and ecologically important. However, reports are usually limited to individual fungal host species or selected groups of associated bacteria, and a more general overview about the distribution and frequency across habitats of such fungus–bacteria associations is still lacking.

Altogether, the currently available data indicate that associations could generally be one important key-factor for the frequent occurrence and global distribution of Mortierellaceae. The ability to grow at temperatures around 0 °C could constitute another key-factor for the global success of this fungal lineage. Mortierellaceae belongs to the most active fungal taxa in snow-covered soil (Kuhnert et al., 2012; Dresch et al., 2019). During winter, they are not only part of the soil microbiota but also have been reported to grow on the soil surface, forming dense under-snow mats, as reported from subalpine forests in Colorado (United States). *In vitro*, these snow-mat fungi (eight unidentified Mortierellaceae spp.) had very high growth rates at −2 °C (Schmidt et al., 2008).

Thus, we aimed to address the following questions with this extensive study on Mortierellaceae: Are environmental conditions and habitat properties influencing the Mortierellaceae distribution? How does snow-cover influence the Mortierellaceae diversity and distribution? And, most interestingly, we wanted to address the question of whether or not Mortierellaceae spp. are associated to bacteria, and if such associations might be species-specific. For addressing these questions, we isolated Mortierellaceae spp. without using antibiotics from a wide range of sites in the Alps, and from both, snow-free and snow-covered soil. We surveyed high-altitude stone pine (*Pinus cembra*) forests, alpine dwarf-willow (*Salix retusa, S. reticulata, Dryas octopetala*) habitats, and alpine bare terrain.

## Materials and Methods

### Sampling and Isolation of Mortierellaceae Isolates

Mortierellaceae pure cultures were isolated from ten different sampling sites in subalpine and alpine habitats of the Austrian Alps at an altitudinal range from 1541 to 2450 m. These included the following habitat types: bare terrain in the glacier forefield, alpine chalk dumps (Kalkschutthalden), alpine dwarf-willow communities (*Salix herbacea, S. reticulata, Dryas octopetala*), high-altitude *Pinus cembra* forests, and subalpine mixed *P. cembra/Picea abies* forests (Table 1). To study seasonal dynamics, at each location sampling was carried out from both snow-covered and snow-free soil. All locations are usually snow-covered in winter (November – May) and snow-free during summer (June – October).

**TABLE 1:** Sampling sites in Austria with information on the origin of isolates the dominating plant communities and sampling time.

Location	Altitude m a.s.l.	Habitat	pH	C/N	Moisture content (%)	Soil organic matter (%)
Obergurgl 2 sites	2280, 2450	Bare soil in the glacier forefield	7.0			1.1 (SF)
Glacier forefield		*Salix herbacea*				4.1 (SC)
Hafelekar	2250	*Salix reticulata, Salix herbacea, Dryas octopetala, chalk dumps*	5.3–6.3	14.8 (SF)	71.8 (SF)	58.3 (SF)
Alpine				13.3 (SC)	54.9 (SC)	34.7 (SC)
Pfitscherjoch	2261	*Salix reticulata, Salix herbacea*	4–4.3	13.51 (SF)	31.3 (SF)	8.9 (SF)
Alpine				12.07 (SC)	29.9 (SC)	12.2 (SC)
Kühtai 3 sites	1842, 1974, 2011	*Pinus cembra*	3.4–3.6	20.69	59.4 (SF)	63.1 (SF)
Sub-alpine					45.3 (SC)	45.2 (SC)
Patscherkofel	2260	*Pinus cembra*	3.5–3.7	22.51	50.3 (SF)	69.1 (SF)
Alpine					55.9 (SC)	70.1 (SC)
Praxmar 3 sites	1541–1814	*Pinus cembra, Picea abies*	3.4–3.9	19.10	37.5 (SF)	37.5 (SF)
Sub-alpine					45.6 (SC)	25.9 (SC)
Haggen	2026	*Pinus cembra*	3.8–3.9		38–47(SF)	27.0 (SF)
Alpine					44–51(SC)	29.0 (SC)

*SC, snow-covered; SF, snow-free.*

Cultivation of Mortierellaceae was carried out either directly from the soil, or using in-growth mesh bags (MBs) containing sterile quartz sand. Mesh bags (4 × 3 cm) were sewed from nylon mesh (72 μm mesh size) and filled with acid-washed, sterilized quartz sand. They were buried for 2 months during the vegetation period, and for the whole snow-covered period (6–8 months) during winter. MBs were stored at −80 °C. For cultivation by direct plating, few particles of soil grains or quartz sand from MBs were plated on agar plates containing potato dextrose agar (PDA) or _LC_A media (Takashima et al., 2019) without antibiotics, and incubated at 10 °C for at least a week. The plates were monitored daily and pure cultures were separated to new plates based on their growth. Several consecutive isolation steps were carried out to obtain fungal pure culture isolates.

### Sequencing of rDNA ITS Regions of Mortierellaceae Isolates

Molecular identification of the obtained fungal isolates was carried out by sequencing the rDNA-ITS region. Colony PCR was performed using aerial mycelium from 7-day-old cultures based on Walch et al. (2016), with the following modifications: The template for the identification of the fungi was prepared by harvesting a little amount of mycelium with sterile water, homogenized and incubated at 85 °C for 15 min. The rDNA-ITS region was amplified by standard primers ITS1 and ITS4 (White et al., 1990). Microsynth AG (Balgach, Switzerland) carried out the sequencing of the PCR products. GenBank accession numbers for all ITS sequences are provided in Supplementary Table 1. Sequences were binned to operational taxonomic units (OTUs) based on 99% sequence similarity using Sequencher 5.2.3. OTUs were used for further analysis. If OTUs with identical species annotation and location inside the phylogenetic tree did not differ in terms of their location distribution and seasonal occurrences, they were summarized to reduce the high number of different Mortierellaceae observed here, which simplifies the accessibility and presentation of the data (especially in the figures).

### Phylogenetic Analysis of Mortierellaceae Isolates

A total of 389 ITS sequences were newly generated in the current study. The obtained sequences were first quality checked, then preliminarily identified based on blastn searches and finally used for phylogenetic analysis. The longer ITS sequences obtained from few isolates were trimmed to avoid misalignment of the species level phylogenetic clade. Along with our ITS sequences, 36 sequences from type material and environmental sequences retrieved from GenBank were included in the alignment. Sequences were then aligned using MAFFT v.7 (Katoh et al., 2017). The alignment was checked using Mega X (Kumar et al., 2018). The best Maximum Likelihood (ML) model was calculated in MEGA X before carrying out an ML analysis. To evaluate branch robustness of trees, Maximum Parsimony based bootstrap analyses were applied. Bootstrap analyses (1000 replicates) were conducted by Subtree-Pruning-Regrafting (SPR) algorithm level 3 in which the initial trees were obtained. For the ML analysis and the bootstrap search, all positions with less than 95% site coverage were eliminated.

Additionally, branch robustness was tested with Bayesian Inference in MrBayes 3.2.6 (Ronquist et al., 2012). For the Markov Chain Monte Carlo (MCMC) analyses, four chains were run for 10 million generations, with trees being sampled every 5000 generations. The analysis was stopped as the convergence diagnostic (average standard deviation of split frequencies) was below 0.05 after 10 million generations. From the 20000 sampled trees (for each of the 2 runs) 25% were discarded as burn-in before summary statistics were calculated (using sump and sumt commands). Diagnostic plots, as well as the convergence diagnostics EES (Estimated Sample Size; min ESS around 10 K) and PSRF (Potential Scale Reduction Factor; 1.000 for all parameters), indicated stationarity. Phylogenetic trees were drawn with Figtree 1.4.4 (Rambaut, 2009).

### Detection and Identification of Mortierellaceae Associated Bacteria

Mortierellaceae pure culture isolates did not show any sign of bacterial contaminations when growing under standard conditions. The presence/absence of associated bacteria was targeted by sequencing of the bacterial 16S rRNA region. The same template as prepared for the fungal colony PCR was used to identify the associated bacteria as well. The PCR amplification of the 16S region was as follows: 25 μl of a PCR mixture containing 2.5 μl of ten-fold buffer S (15 mM MgCl_2_, 100 mM Tris, 500 mM KCl), 2.5 μl of 10% BSA, 1 μl of primers 8f and 1492r, each (10 mM), 0.5 μl of dNTPs (10 mM each), 1 μl of DNA template and 0.15 μl of LabQ Taq-pol (5 units/μl), and 16.35 μl of sterile PCR water. The bacterial PCR cycling conditions were according to Takashima et al. (2018). PCR products were sequenced using the primer 27F by Microsynth AG (Balgach, Switzerland). All sequences obtained in the study are available in GenBank (Supplementary Table 1). Closely related sequences were downloaded from GenBank. To identify the bacteria on a species level, the threshold was set to 99% sequence similarity. Sequences were aligned, and phylogenetic analyses were carried out in Mega X. A Maximum likelihood analysis was carried out based on the best model (Kimura 2-parameter model) as suggested by Modeltest. For the branch robustness Maximum parsimony bootstrapping analyses was done with 1000 replicates with 95% site coverage was eliminated. Bacterial sequences were binned to OTUs based on 99% sequence similarity using Sequencher 5.2.3.

### Soil Properties

Moisture content was determined based on the weight loss of the sieved soil sample when incubated at 105 °C overnight. The soil organic matter (SOM) was determined based on loss of ignition at 550 °C for 7 h. The pH of the samples was measured with 0.01 M Calcium chloride (CaCl_2_) potentiometrically. The soil properties were measured as suggested by Kuhnert et al. (2012). The carbon and nitrogen element analysis was performed by an organic elemental analysis flash combustion instrument (Thermo Fischer Scientific, Austria).

### Statistical Analysis

All statistical analyses of the data were performed in R 4.0.2 (R Core Team, 2020). For alpha diversity analysis, package vegan v2.5-6 (Oksanen et al., 2019) was used. In order to test for influences of environmental variables (pH, C/N, moisture, soil organic matter) and environmental factors [season (snow-cover vs. snow-free) and habitat (alpine bare terrain, dwarf-willow communities, *P. cembra* forest)] permutational multivariate analysis of variances was applied on a Bray–Curtis distance matrix using adonis function (999 permutations). For clustering of fungal occurrences, bacterial associations, and Mortierellaceae–bacterial pairs as well as the clustering of Mortierellaceae species based on their association to *Pseudomonas* OTUs, several clustering algorithms were tested and compared. For fungal clustering, the distance matrix was based on Bray–Curtis dissimilarity. All other variables were inherently dependent on the distribution of the Mortierellaceae species and on the total number of observations in this sample group. Therefore, we calculated the distance between two sample groups as the number of shared observations normalized by the maximum number of possible shared observations. For example, if there were two different fungus–bacterium pairs observed in location A and five different fungus–bacterium pairs in location B and only one of those associations would be found in both locations, the distance between location A and B would be 0.5. For each clustering, different clustering algorithms were applied. For all clusterings, the ward algorithm best represented all other algorithms and, consequently, it was chosen for display. Networks were drawn from an edgelist containing all observed Mortierellaceae*–*bacteria associations using R package igraph (Csardi and Nepusz, 2006). The dataset is available as Supplementary Table 2.

Our dataset contained several observations that were only made once. While for alpha diversity calculations, these have not been disregarded, for visualization, these one-time occurrences have been omitted from display.

## Results

### Mortierellaceae Composition Is Diverse, Widespread, and Affected by Habitat, Location, and Snow-Cover

**FIGURE 1:**
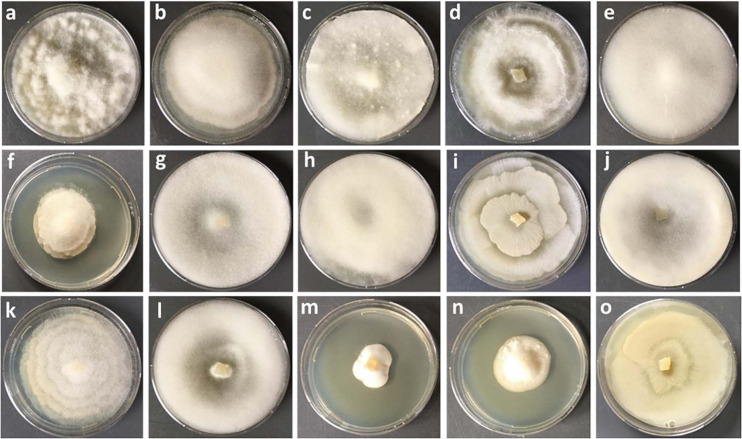
Culture characteristics of different species of Mortierellaceae isolated on PDA. **(a)** *Podila verticillata*, **(b)** *Linnemannia elongata*, **(c)** *M. pseudozygospora* (*s.l.*), **(d)** *M. angusta* (*s.l.*), **(e)** *P. minutissima*, **(f)** *M. solitaria*, **(g)** *M. zonata* (*s.l.*), **(h)** *L. hyalina*, **(i)** *L. gamsii*, **(j)** *M. gemmifera* (*s.l.*), **(k)** *Entomortierella parvispora*, **(l)** *M. bainieri* (*s.l.*), **(m)** *M. globalpina* (*s.s.*), **(n)** *M. alpina* (*s.s.*), **(o)** *L. exigua.*

**FIGURE 2:**
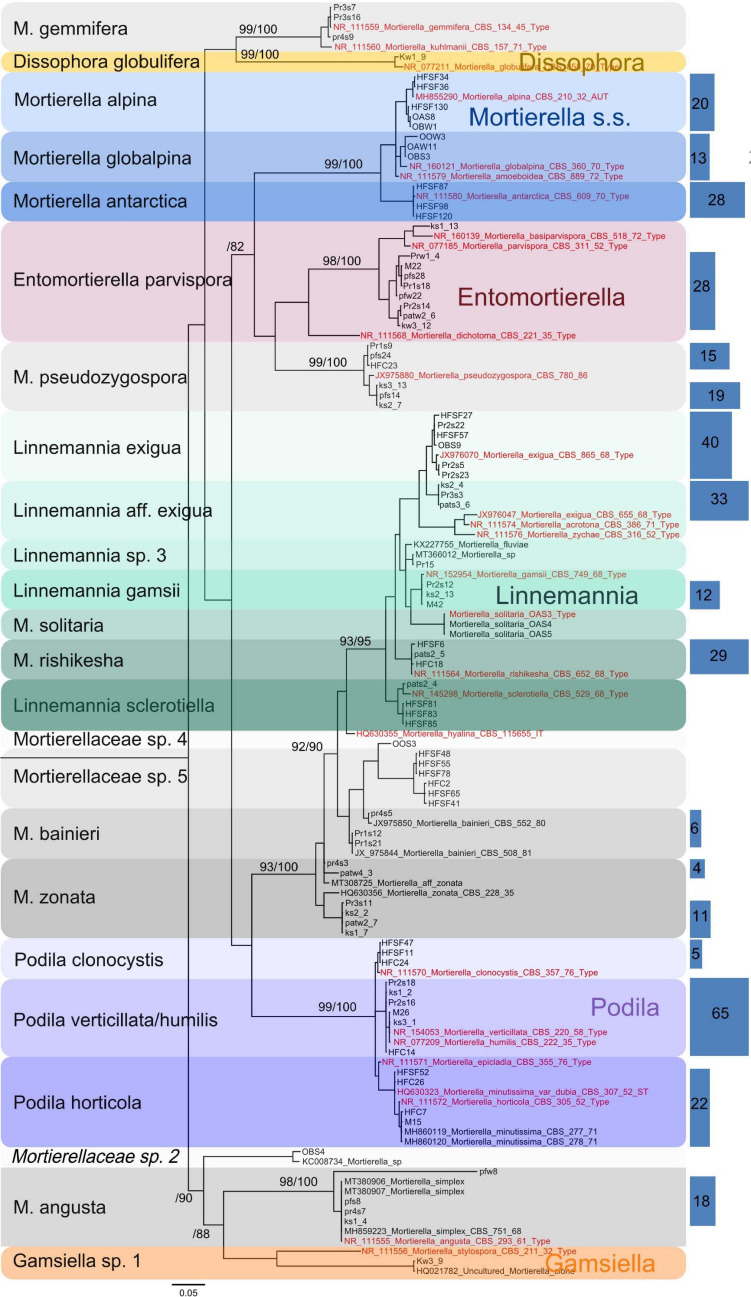
Phylogenetic tree of the Mortierellaceae species isolated in this study (*n* = 389 isolates) (loglikelihood –3810.59) based on rDNA-ITS sequences. The isolates fall into 24 well-supported clades. For branches close to the origin, branch ≥70 is shown above the branches as Bayesian posterior probabilities/Parsimony-based bootstrap values. Sequences originating from type specimens are highlighted in red. In order to reduce the size of the tree, all isolates’ sequences were binned to 99% OTUs and a representative number of sequences for each OTU were shown. Blue rectangles on the right illustrate the total number of isolates represented by the sequences they cover (same height). The abundance of single sequences that are directly represented in the tree (*n* = 1) is not illustrated by rectangles. Rectangle areas are proportional to each other and to isolate abundance, which is also given in the rectangles.

In this study, a total number of 389 Mortierellaceae pure cultures were isolated and analyzed (Figure 1). Phylogenetically, these clustered into 24 terminal clades based on their rDNA-ITS sequences, representing OTUs with ≥99% sequence similarity (Figure 2). The majority of our isolates could unambiguously be identified based on available type sequences (19 clades: 348 isolates). *Podila humilis* and *P. verticillata* could not be differentiated based on their ITS sequences and were summarized to one species complex (Figure 2). Isolates, that could not be identified fell into five distinct clades. Those likely represent new species that remain to be described, and we addressed them as Mortierellaceae sp. 1–5 in our study.

**FIGURE 3:**
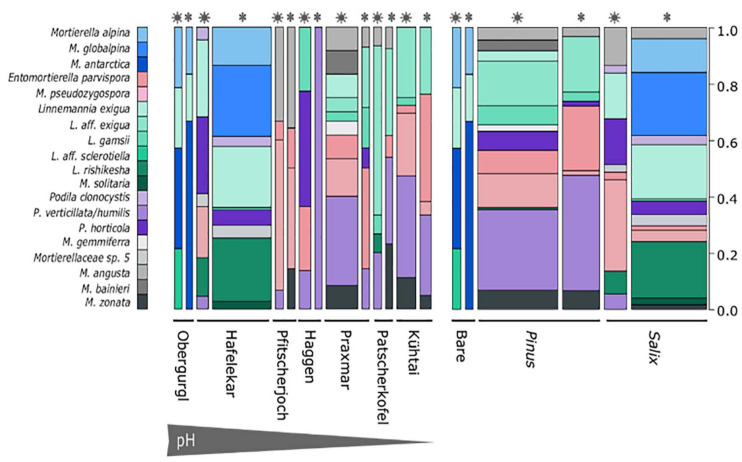
Mortierellaceae species distribution across sampling sites **(left)** and habitats **(right)**. Snowflakes and stars indicate whether the sample, from which the strain was isolated, was covered with snow or not. Species represented by one single species were excluded from the illustration. Bar width is proportionate to the sample size of the sample group in the overall data set. *M. alpina, M. globalpina* and *M. antarctica* represent *Mortierella* s.s., all other *Mortierella s.l*.; E., *Entomortierella*; L., *Linnemannia*; M., *Mortierella*; P., *Podila*.

Mortierellaceae spp. were isolated from all habitats and occurred in both snow-covered and snow-free soil. The numbers of both isolates and species isolated per location varied strongly (Table 2). The species composition of Mortierellaceae differed between sampling sites (Figure 3), especially between dwarf-willow habitats and *P. cembra* forests (33% variance, p_Adonis_ = 0.031, Supplementary Figure 1). Moreover, Mortierellaceae composition was also correlated to soil pH (41% variance, p_Adonis_ = 0.001), and to a minor extent to C/N ratio (20% variance, p_Adonis_ = 0.063). Furthermore, snow-cover (seasonality) seemed to influence the species composition (Figure 3).

**TABLE 2:** Overview of Mortierellaceae composition at different locations, in different habitats, and in snow-covered and snow-free soil.

	Depth	Richness	Shannon	Evenness	Depth/Richness
**Obergurgl**	28	6	1.45	0.81	5
**Hafelekar**	133	11	1.98	0.83	12
**Pfitscherjoch**	29	5	1.26	0.78	6
**Haggen**	35	4	1.26	0.91	9
**Praxmar**	75	12	2.21	0.89	6
**Patscherkofel**	29	8	1.59	0.76	4
**Kühtai**	60	9	1.75	0.80	7
**Bare terrain**	28	6	1.45	0.81	5
***Pinus***	199	17	2.16	0.76	12
***Salix***	162	14	2.26	0.86	12
**Snow-free**	188	21	2.53	0.83	9
**Snow-covered**	201	19	2.58	0.88	11

*Depth, total number of isolates; Richness, number of different Mortierellaceae species detected; Shannon, Shannon index of Mortierellaceae community; Evenness, Pilou’s evenness [Shannon/ln(richness)]. Depth/richness expresses the average number of isolates belonging to the same species isolated in a sample group.*

Independent of the species’ abundances, that is the number of isolates at a site or in a habitat, the majority of species were isolated from a single location or showed habitat specificity. *Mortierella alpina* (*s.s.*) and *P. clonocystis* were isolated from different locations but exclusively found in dwarf-willow habitats. *M. antarctica* (*s.s.*), *Linnemannia rishikesha*, and an unknown Mortierellaceae sp. 5 were obtained only from the dwarf-willow habitats at one calcareous site (Hafelekar). *Mortierella globalpina* (*s.s.*) and *M. solitaria* (*s.l.*) were isolated solely from alpine bare terrain in the glacier forefield (Obergurgl). Other species are typical for *P. cembra* forests: With few exceptions, *L. gamsii, Entomortierella parvispora, M. bainieri* (*s.l.*), *M. gemmifera* (*s.l.*) and *M. zonata* (*s.l.*) were typically isolated from this habitat. The latter two species were exclusively obtained from Praxmar. However, we detected also generalist species: For *M. angusta* (*s.l.*), *P. horticola*, and *M. pseudozygospora* (*s.l.*) no location or habitat specificity could be detected.

The genus *Linnemannia* appears to be especially abundant in subalpine and alpine soil habitats. *Linnemannia exigua* was the most abundant species observed in our dataset (73 isolates). According to the ITS phylogeny, almost half of these isolates (33 isolates) formed a subclade (Figure 2). Interestingly, all isolates belonging to the subclade *L. affinis (aff.) exigua* were isolated solely from *P. cembra* forests (Figure 3). The isolates belonging to clade *L. exigua* were isolated across different locations and habitats but showed a higher abundance in dwarf-willow habitats.

*Linnemannia sclerotiella* originated from a snow-free *P. cembra* forest soil, while three sister group isolates were obtained from a snow-covered dwarf-willow habitat (Hafelekar). According to the ITS-based phylogeny, this sister-group might represent a new species. A higher sampling size would be necessary for confirming the environmental preferences of this species (Figure 3).

The second largest group of isolates is represented by the genus *Podila* (*P. humilis/verticillata* clade; 65 isolates). They, too, were found across different locations, but were most frequently isolated in *P. cembra* forests (Figure 3).

Several species were isolated once or a few times, only, e.g., *E. basiparvispora, Dissophora globulifera, Gamsiella sp. 1*, and *M. solitaria* (*s.l.*). Consequently, their distribution could not be determined.

While there was little difference in Mortierellaceae compositions between seasons in the alpine sites Obergurgl and Pfitscherjoch, the Mortierellaceae species compositions of the forest sites exhibited a clear seasonal variation (snow-covered soil vs. snow-free soil) (Figure 3). Especially the lowest site in terms of altitude, Praxmar, appeared to have a specific summer and a specific winter Mortierellaceae community. Some *Mortierella s.l.* species, *M. solitaria, M. bainieri*, and *M. gemmifera*, were only isolated from snow-free soil types. *M. antarctica* (*s.s.*) occurred only in snow-covered soil (Figure 3).

The Mortierellaceae richness of a sampling site ranges between 4 and 12 species per site. The richness increases with decreasing altitude and is highest in *P. cembra* forests. The richness did not differ significantly between dwarf-willow habitats and *P. cembra* forests. There were no significant differences in richness between snow-covered and snow-free soil (Table 2). The total number of isolates differed between sampling sites (Table 2), although the sampling effort was comparable. The total number of isolates obtained from one sampling site influenced Mortierellaceae richness, that is number of Mortierellaceae OTUs, and Mortierellaceae diversity, here expressed by Shannon index (*r* = 0.76, *p* = 0.049). The calcareous alpine site with dwarf-willow vegetation, Hafelekar, exhibited unique features compared to all other sampling sites. Given a high number of 133 isolates obtained from this site, only 11 different species were detected. In contrast, at the *P. cembra* site Patscherkofel and in bare glacier foreland terrain (Obergurgl), where not even 30 isolates were obtained, eight and six different species, respectively, were detected (Table 2). In terms of both richness and diversity, there was no pattern detectable among locations, despite the compositional pattern following soil pH and C/N ratio. For the majority of the sampling sites, the Mortierellaceae composition was uneven, with the exception of the pure *P. cembra* plantation at Haggen (Figure 3).

### Mortierellaceae spp. Are Frequently Associated With Bacteria

**FIGURE 4:**
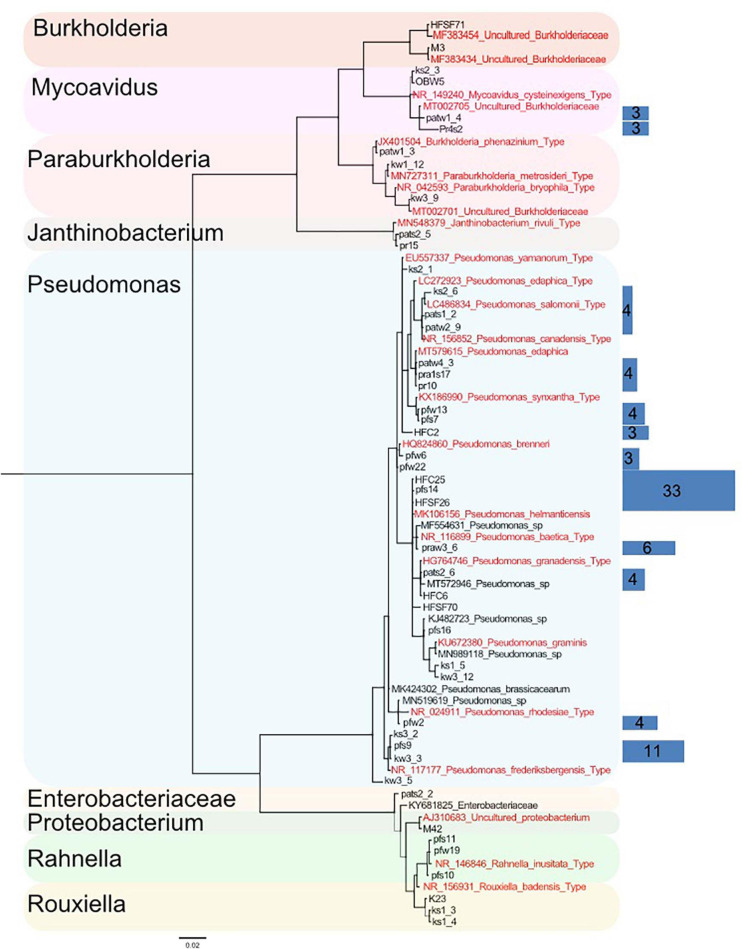
Phylogenetic tree of a representative subset of Mortierellaceae-associated bacteria based on their 16S region (log-likelihood –3459.28). A total of 126 bacteria were associated to Mortierellaceae isolates. They were binned into operational taxonomic units (OTUs) based on their 16S sequence (99% sequence similarity). From each OTU, a representative number of sequences were chosen, from which the phylogeny was constructed. The detected bacteria fall into nine lineages. Relationships are generally well-supported. Support values [Bayesian posterior probabilities (BS)/Parsimony-based bootstrap values (BPP)] are provided for the nine major lineages besides the respective nodes. Thin lines represent relationships with BS and/or BPP < 80%. Sequences originating from type specimens are highlighted in red. Blue rectangles on the right illustrate the total number of isolates represented by the sequences they cover (same height). The abundance of single sequences that are directly represented in the tree is not illustrated by rectangles (*n* = 1). Rectangle areas are proportional to each other and isolate abundance, which is also provided as numbers in the rectangles.

Out of 389 Mortierellaceae isolates studied here, 126 were found to be associated with bacteria (32%), all of them Proteobacteria, and all Mortierellaceae species independently isolated at least three times were associated with bacteria (*n* = 18). In order to illustrate their relationship and diversity, a phylogenetic tree of the bacteria associated with the isolated strains was calculated based on their full-length 16S sequence. According to the terminal clades of the resulting tree (Bootstrap values and Bayesian Posterior probabilities ≥70%), the associated bacteria belonged to seven different genera (Figure 4). Two bacteria could not be identified on a genus level: one is a Proteobacterium, and the other is a member of Enterobacteriaceae. The discrimination among closely related bacteria was difficult due to their high sequence similarity, especially for *Pseudomonas* species. Hence, bacterial sequences were binned into OTUs (99% sequence similarity) and a representative number of sequences for each OTU were included in the phylogenetic tree (Figure 4). Although the phylogeny was generally well supported, for some OTUs (5/20 OTUs, Supplementary Table 2), their representative sequences were not clustered in direct neighborhood. Therefore and as this study did not target phylogenetic questions, we used well supported lineages for further analysis.

The vast majority of bacteria associated with Mortierellaceae isolates represented species of the genus *Pseudomonas* (96/126 = 76%, Table 3). Moreover, we detected species of the genera *Mycoavidus* (10/126), *Rahnella* (7/126), *Burkholderia* (3/126), *Paraburkholderia* (3/126), *Rouxiella* (3/126), and *Janthinobacterium* (2/126).

**TABLE 3:** Bacterial associations of Mortierellaceae species.

	*Burkholderia*	*Paraburkholderia*	*Mycoavidus*	*Pseudomonas*	*Janthinobacterium*	*Rahnella*	*Rouxiella*	Enterobacteriaceae	Proteobacterium	Freq. total	Freq. bac	Bac [%]	*Pseudomonas* [%]	Bac diversity
*Entomortierella parvispora*	0	2	0	7	0	0	0	0	0	28	9	32	78	2
*Linnemannia* aff. *exigua*	0	0	5	10	0	0	0	0	0	33	15	45	67	2
*Linnemannia exigua*	1	0	2	17	0	0	0	0	0	40	20	50	85	3
*Linnemannia gamsii*	0	0	1	3	0	0	0	0	1	12	5	42	60	3
*Mortierella alpina*	0	0	0	0	0	0	0	0	0	20	0	0		0
*Mortierella antarctica*	0	0	0	1	0	0	0	0	0	28	1	4	100	1
*Mortierella globalpina*	0	0	0	0	0	0	0	0	0	13	0	0		0
*Podila horticola*	1	0	0	4	0	1	0	0	0	22	6	27	67	3
*Podila humilis/verticillata*	0	0	2	14	0	0	1	0	0	65	17	26	82	3
*M. angusta* (*s.l.*)	0	1	0	7	0	4	0	1	0	18	13	72	54	4
*M. pseudozygospora* (*s.l.*)	0	0	0	9	0	1	2	0	0	34	12	35	75	3
*M. rishikesha* (*s.l.*)	0	0	0	10	1	0	0	0	0	29	11	38	91	2
*M. zonata* (*s.l.*)	0	0	0	3	0	1	0	0	0	15	4	27	75	2

*Freq. total, total number of Mortierellaceae isolates belonging to this species; Freq. bac and bac [%], total number and percentages of isolates associated to bacteria, respectively; *Pseudomonas* [%], percentage of bacteria annotated as *Pseudomonas*; Bac diversity, number of different bacterial genera detected in the respective Mortierellaceae species.*

The frequency of associations depended on the fungal species (Supplementary Figure 2). For some Mortierellaceae species, around half of their isolates were associated with bacteria, indicating there was no selection in terms of whether or not such fungus was associated with a bacterium (Table 3). For other species, associations were rare (<25%). For *M. alpina* and *M. globalpina* (both *s.s.*), no bacterial associations were detected. In contrast, *M. angusta* (*s.l.*) was more frequently associated with bacteria than the other Mortierellaceae (>75%). Generally, Mortierellaceae species with a representative number of both isolates and bacteria-associated isolates were associated with more than one bacterial genus (Table 3).

**FIGURE 5:**
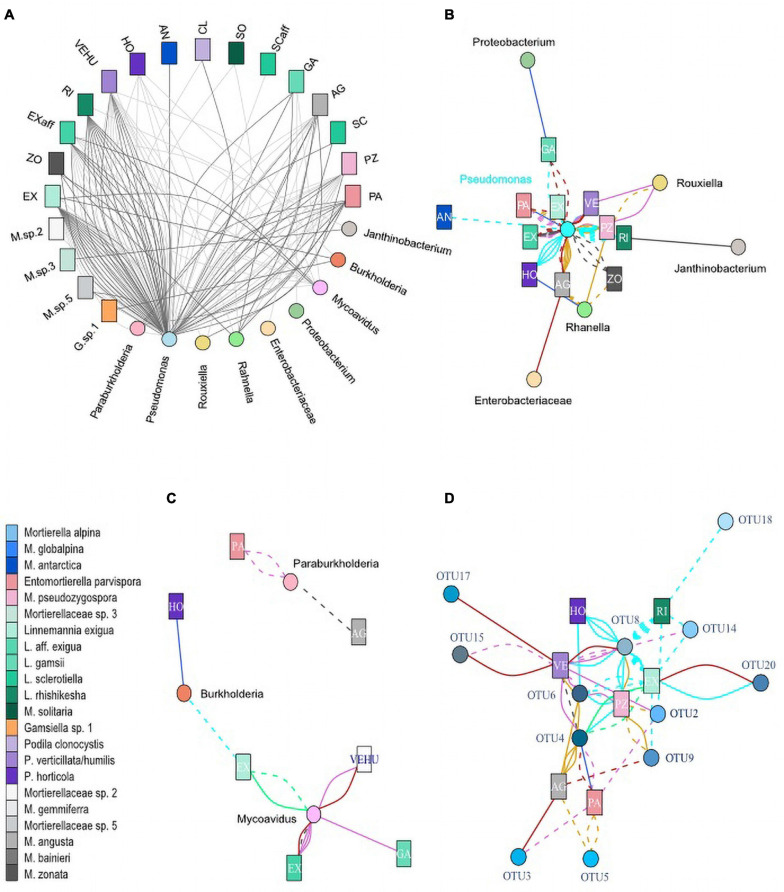
Co-occurrences of Mortierellaceae isolates and bacteria. Each line represents an isolated Mortierellaceae strain with an associated bacterium. **(A)** Seasonal dependence of co-occurrences on snow-cover. Dark gray lines, isolate was obtained from snow-free soil; light gray lines, isolate was obtained from snow-covered soil. **(B)** Co-occurrences of isolated Mortierellaceae strains and *Burkholderiales*. **(C)** Co-occurrences of isolated Mortierellaceae strains and Pseudomonadales and Enterobacteriales. **(D)** Co-occurrences of isolated Mortierellaceae strains and *Pseudomonas* OTUs. For panels **(B–D)**, line colors indicate the location of strain isolation: cyan, Hafelekar; royalblue, Haggen; orchid, Kühtai; springgreen, Obergurgl; purple, Patscherkofel; goldenrod, Pfitscherjoch; firebrick, Praxmar. Solid lines, isolate was obtained from snow-covered soil; dashed lines, isolate was obtained from snow-free soil. AG, *M. angusta* (*s.l.*); AL, *M. alpina* (*s.s.*); AN, *M. antarctica* (*s.s.*); BA, *M. bainieri* (*s.l.*); CL, *Podila clonocystis*; Ex, *Linnemannia exigua*; Exaff, *L.* aff. *exigua*; GA, *L. gamsii*; GL, *M. globalpina* (*s.s.*); HO, *P. horticola*; M. sp. 5, *Mortierellaceae* species complex 5; PA, *Enteromortierella parvispora*; PZ, *M. pseudozygospora* (*s.l.*); RI, *L. rishikesha*; SCaff, *L.* aff. *sclerotiella*; SC, *L. sclerotiella*; SO, *M. solitaria* (*s.l.*); VEHU, *P. verticillata/humilis*; ZO, *M. zonata* (*s.l.*).

The dependency of Mortierellaceae species distribution on the environmental factors, location, habitat and season, was inherently reflected in the distribution of associations to bacteria (Figure 5 and Supplementary Figures 1, 3, 4). However, Mortierellaceae*–*bacteria pairs were not randomly distributed across the occurrence spectrum of Mortierellaceae species. They, too, were influenced by those environmental factors. *Pseudomonas* (*Pseudomonadales*) was detected in association with Mortierellaceae species across all locations (Supplementary Figures 3, 4).

Associations of Mortierellaceae species to members of the Enterobacterales were limited to both a low number of observations and locations (Supplementary Figure 2). *Rahnella* was found associated with *P. horticola, M. pseudozygospora* (*s.l.*), *M. zonata* (*s.l.*), and *M. angusta* (*s.l.*) in Pfitscherjoch (dwarf-willow) and Haggen (*P. cembra*) (Supplementary Figure 4). *Rouxiella* was found associated in the sampling sites Pfitscherjoch (dwarf-willow) and Kühtai (*P. cembra*). Associations were limited to *P. humilis/verticillata* and *M. pseudozygospora* (*s.l.*).

In addition to Pseudomonadales and Enterobacterales, we found Burkholderiales members associated to Mortierellaceae species. *Mycoavidus*, which was the second frequent bacterial genus associated with Mortierellaceae, was detected in Obergurgl and all *P. cembra* forests, except Haggen (Supplementary Figures 2, 4), which might be due to sampling size. *Mycoavidus* was associated with *L. exigua, L. aff. exigua*, and *P. humilis/verticillata* (Figure 5C). *Burkholderia* was found in Hafelekar and also in Haggen. *Paraburkholderia* were detected only in *P. cembra* forests, Kühtai, and Patscherkofel. They were mainly associated with *E. parvispora* (Figure 5C). Against the odds, *L. aff. exigua, L. exigua, P. humilis/verticillata*, and *E. parvispora* were frequently associated to bacteria from different Proteobacteria orders (Figures 5A,C). *Mortierella angusta* (*s.l.*), *M. zonata* (*s.l.*), *M. pseudozygospora* (*s.l.*), and *L. rishikesha* (the latter appeared associated only with *Pseudomonas*) were not found associated to Burkholderiales (Figure 5B and Supplementary Figures 2, 3).

Altitude not only affected the occurrences of associations but generally impacted on the association patterns between Mortierellaceae and bacterial lineages (Figures 5B,C). *Entomortierella parvispora, M. pseudozygospora* (*s.l.*), and *M. zonata* (*s.l.*), who were all detected across several locations (Figure 3), were associated to bacteria only in those locations at higher altitude and associations were usually absent in Praxmar, which was the location with the lowest altitude in this dataset (Table 1 and Figure 5). The exception of this rule was *P. humilis/verticillata*, for which it was the other way around. *Linnemannia exigua*, who was detected across all habitats (Figure 3), was associated with *Burkholderia* only at high altitude locations [e.g., Obergurgl (bare ground)] (Figure 5).

Altitude appeared to have a stronger impact on Mortierellaceae*–*bacteria associations compared to habitat (dwarf-willow community vs. *P. cembra* forest), as *E. parvispora* and *M. zonata* (*s.l.*) were both isolated more frequently from *P. cembra* forests than dwarf-willow habitats (Figure 3), while associations to bacteria were found equally frequently in both habitats (*M. zonata* (*s.l.*)*: P. cembra* = 2/13, dwarf-willow = 2/3; *M. zonata* (*s.l.*)*: P. cembra* = 7/25, dwarf-willow = 2/3) (Figure 5).

In addition to altitude/location and habitat, also the season influenced the associations between Mortierellaceae and bacteria (Figure 5A). While there was no seasonal effect on the distribution of *M. angusta* (*s.l.*), *M. zonata* (*s.l.*), *P. horticola*, and *E. parvispora* (Figure 3), the snow-cover affected whether or not these species were found associated with bacteria or to which bacterial genera they were associated. Under snow-cover, *M. angusta* (*s.l.*) was more frequently associated with *Rahnella* (3/4) than with *Pseudomonas* (2/7), while in snow-free sites, the pattern was reversed. *M. zonata* (*s.l.*) (4/4) and *E parvispora* (8/9) were more frequently associated with bacteria under snow-cover compared to snow-free conditions. For *P. horticola*, associations were detected only in snow-free conditions (6/6) (Figure 5A).

As *Pseudomonas* was associated with all Mortierellaceae species investigated here, and these associations were detected across all locations, we asked for a potential specificity on 99% bacterial OTU level (Supplementary Table 2). Strikingly, almost all Mortierellaceae species with more than one association to *Pseudomonas* were located in one big branch, including *Podila* and *Linnemannia*, carrying the highest diversity and most closely related species. With regards to *Pseudomonas* OTUs, no Mortierellaceae species specificity could be detected (Figure 5D). However, clustered by their association to *Pseudomonas* OTUs, especially the phylogenetic relationship among *Linnemannia* species, but also the placement of *P. clonocystis* and *P. humilis/verticillata* (Figure 2) was loosely conserved (Supplementary Figure 5).

## Discussion

### Taxonomy and Nomenclature of Mortierellaceae

Mortierellaceae species have been extensively studied (Linnemann, 1941; Milko, 1974; Gams, 1977; Wagner et al., 2013) but the evolutionary relationship remained unresolved. A recent study now could resolve the phylogeny of the Mortierellaceae based on multigene phylogenetics and phylogenomics (Vandepol et al., 2020). However, several of our isolated Mortierellaceae have not yet been recombined into new genera, and their exact taxonomic position still needs to be resolved, which is far beyond the scope of this paper. In order to clearly differentiate between *Mortierella* species that have not been placed into the new phylogeny and those that have, we will indicate the former by *sensu lato* (*s.l.*) and the latter by *sensu stricto* (*s.s.*). Species identification of Mortierellaceae, especially for the genera *Mortierella* (*s.s.*), *Podila* and *Linnemannia*, is not always possible by ITS sequence due to their high sequence similarity (Vandepol et al., 2020). We used OTUs with 99% sequence identity for our fungal isolates, which can then clearly be differentiated, and were in agreement with most recent taxonomy. Here, we used phylogenetic analysis and information solely in order to show the diversity of our isolates and to illustrate and support their membership to existing and likely undescribed genera, respectively. For these purposes and for constructing species representing-clades, the sequence of the ITS region usually suffices.

### Factors Shaping Mortierellaceae Communities

The main aim of our study was to understand the factors shaping the distribution and diversity of Mortierellaceae and their associations with bacteria in the alpine region. The prevalence and geographical distribution of species belonging to this family suggests that these fungi are playing a key role in the soil microbiota of alpine and subalpine habitats. Our results show that Mortierellaceae species are widely distributed and quite abundant in alpine and subalpine forests. Species distribution across all the sampling sites showed habitat specificity, suggesting that habitat properties determined Mortierellaceae communities. Some of the obtained species were exclusively found in the bare terrain of the glacier forefield sites, others exclusively in *P. cembra* forests, clearly indicating that these Mortierellaceae species are habitat-specific. For example, *M. solitaria* (*s.l.*) and *M. globalpina* (*s.s.*) were isolated from bare terrain, whereas *Entomortierella parvispora, Linnemannia gamsii, M. gemmifera* (*s.l.*), *M. bainieri* (*s.l.*), and *M. zonata* (*s.l.*) were isolated from *P. cembra* forests. In the phylogenetic tree calculated from ITS sequences, these latter three species were located in the *Linnemannia* branch. They were not included in the study of Vandepol et al. (2020). According to Wagner et al. (2013) their taxonomic placement needs revision (*M. gemmifera, M. zonata*) and both *M. gemmifera* and *M. zonata* were placed into group 7, from which several species have been recombined into *Linnemannia* by Vandepol et al. (2020). Taking together this information and the close phylogenetic relation between *Linnemannia* and *Entomortierella* (Vandepol et al., 2020), our data indicate a preference of *P. cembra* forests of some members of these genera. Further research on these potential relations is necessary, which are beyond the scope and results of this study.

In addition, altitude impacted on both, the richness and the species composition of Mortierellaceae communities. This is in line with data reported for the soil and rhizosphere microbial diversity of *Ranunculus glacialis* habitats along a high-altitudinal gradient. Mortierellaceae communities were altitude dependent, changed with altitude, and had the highest abundances in the nival zone (Praeg et al., 2019). However, the factors habitat and altitude also imply drastic changes in environmental factors such as soil pH, soil organic matter (SOM), duration of snow-cover, and C/N, which are characteristic for habitat type and sampling sites (Siles and Margesin, 2016).

In our dataset soil pH showed a pronounced effect on the Mortierellaceae communities in alpine habitats. Certain species of Mortierellaceae colonize acidic soils, whilst other species prefer calcareous soil with neutral pH (Yamanaka, 2003; Ning et al., 2020). The investigated dwarf-willow habitats had a higher pH variation and plant diversity compared to *P. cembra* forests, thus explaining the higher diversity and variation in Mortierellaceae species composition. Our dataset also suggested that C/N ratio and seasonality influence the distribution of Mortierellaceae species. This further explains the abundance and species richness at the studied sites and habitats (Table 2).

Seasonal dynamics have also been reported to impact on the microbial communities, including those in alpine habitats (Bardgett et al., 2005; Žifčáková et al., 2016; Dresch et al., 2019) but this has never been investigated for distinct fungal lineages, like Mortierellaceae. The presence of snow-cover is an important factor for soil physiological processes and soil organic matter (SOM) build-up. Snow-cover favors undisturbed saprobial activity under constant conditions, with reduced competition or predation. In contrast, rhizosphere processes related to the photosynthetic activity of plants are highly important during the vegetation processes. They are the first step for manifold synergistic interactions in the soil, and they are also known to be crucial for SOM formation in boreal forests (Clemmensen et al., 2013), which is also reflected by the high values of SOM in the *P. cembra* forests investigated here. It is evident with our dataset that the seasonal changes did influence the distribution of Mortierellaceae species in their given habitats. For instance, the *Mortierella s.l.* species *M. solitaria, M. bainieri*, and *M. gemmifera*, were only isolated from snow-free soil, whereas *M. antarctica* (*s.s.*) was isolated only from snow-covered soil. As argued above, these former three species need taxonomic reclassification and might be members of the genus *Linnemannia*. Their isolation from solely snow-free and snow-covered soil, respectively, might provide another ecological distinction criteria between the genera *Mortierella* (*s.s.*) and *Linnemannia*.

Several functions have been attributed to Mortierellaceae: they are saprobial soil fungi able to grow under nutrient-poor (oligotrophic) conditions (Bergero et al., 1999) and at high elevations (Schmidt et al., 2012), but are also known as plant endophytes with a dual lifestyle (Zhang et al., 2020). The species-specific physiological features of individual *Mortierella* species are probably contributing to habitat preferences and seasonal dynamics. The patterns by location, habitat and season detected here among Mortierellaceae genera across species based on ITS sequence similarity support their placement based on phylogenetics and phylogenomics (Vandepol et al., 2020) and indicates a potential phylogenetic conservation of some ecological features among species within different genera.

### Mortierellaceae Species Are Regularly Associated With Bacteria

Our expectation was that Mortierellaceae species are associated with different taxa of bacteria, and that such associations are widespread in nature. We detected associated bacteria in almost all our frequently isolated Mortierellaceae species. On average, >30% of the isolates within a species (*n* = 19) were associated to a bacterium, indicating that these associations were facultative. In general, fungi and bacteria co-exist in the environment. Some bacteria can colonize hyphae and move by gliding on the hyphal surface (Simon et al., 2017), or they can invade fungi and dwell inside the fungus (Deveau et al., 2018). Due to this close co-habitation of bacteria and fungi in the soil, the associations between Mortierellaceae and bacteria observed here might seem like artifacts. The possibility of random co-isolations of Mortierellaceae and bacteria during the fungal isolation by direct plating cannot be excluded, but seems unlikely to us for several reasons: (i) Random associations would be taxonomically more diverse and not limited to Proteobacteria as observed here. This is underpinned by the fact that the vast majority of soil bacteria are usually not part of this taxonomic group. (ii) Random associations would not follow environmental trajectories. (iii) Random associations would not show a species-specific pattern (Table 3). Although fungal hyphae are known as bacterial highways (Simon et al., 2017; Jiang et al., 2021), we did not detect any bacterial species known as frequent passengers of *Fusarium* and *Chaetomium* (e.g., *Variovorax soli*, *Olivibacter soli*, *Acinetobacter calcoaceticus*, and *Stenotrophomonas*, *Achromobacter* and *Ochrobactrum*) (Simon et al., 2015) in our Mortierellaceae strains, supporting the specificity of fungal–bacterial associations.

We were intrigued by the fact that the proportion of bacteria-associated isolates varied depending on the particular Mortierellaceae species. Some species almost never had associated bacteria, despite a high number of isolates (Table 3). For others, such as *M. angusta* (*s.l.*), almost 80% of all isolates were associated with bacteria. In addition, to an apparent preference of the fungal species in terms of bacterial associations, the environmental conditions, including season and habitat, affected Mortierellaceae*–*bacterial pairs. For most Mortierellaceae species, higher habitat altitude resulted in a higher number of bacterial associations, indicating that associations might provide an advantage under these environmental conditions.

In our dataset, *M. alpina* and *M. globalpina* (both *s.s.*) were never found in association with bacteria. This is in contrast to a study from Kai et al. (2012) reporting an association of *M. alpina* and endobacteria. In our study, those species were isolated only from Obergurgl (alpine bare terrain in the glacier forefield) and in the case of *M. alpina*, from Hafelekar (dwarf-willow habitat). It is possible that these species did not form any associations with bacteria due to the environmental factors given in the specific habitat/location. Further studies involving *M. alpina* and *M. globalpina* isolates from a range of different locations are necessary to address this question.

From an ecological perspective, the associations between Mortierellaceae species and bacteria and their potential functions are very interesting. It is also interesting to ask for benefits and burdens for both the fungus and the bacterium for establishing an association and situations, in which these might provide a selective advantage, such as the exchange of metabolites in nutrient limited conditions. Until now, research has been focused on specific, endosymbiotic bacterial lineages. For a more mechanistic understanding of these associations, further studies analyzing numerous isolates from different environmental conditions and screening for a broad range of bacteria, including co-cultivation studies are necessary.

### Mortierellaceae Is Associated to Different Bacteria

*Pseudomonas* was the most widespread and most frequent bacterial taxon associated to Mortierellaceae in this study. Moreover, Mortierellaceae species appeared promiscuous in terms of *Pseudomonas.* However, we observed a phylogenetically conserved clustering of species belonging to *Podila* and *Linnemannia* by *Pseudomonas* OTUs (Supplementary Figure 5). Only *M. angusta* (*s.l.*) and *E. parvispora*, which were the two species with the highest number of exceptions from the rule in our study, did not follow the congruency in *Pseudomonas* clustering and phylogenetic relationships. Although being a weak indication, we consider a potential co-evolution as an interesting option, which should be mentioned as it might be worth further investigations. The phylogenetically conserved clustering could indicate that these fungal–bacterial associations have provided an evolutionary advantage. Independent of co-evolution, the association to *Pseudomonas* might be advantageous in alpine areas. Complete genome sequence of *P. frederiksbergensis*, one of our frequently detected *Pseudomonas* OTUs (Figure 1), revealed that this bacterium owns the genetic basis for survival at high altitudes (Kumar et al., 2019), and efficient lipid metabolism. Cold adaptation and UV protection are important traits for survival at high altitudes and could possibly be provided by the bacterium in exchange for lipids, which are usually abundantly produced by Mortierellaceae species (Sakuradani et al., 2009). A focused, experimental approach is needed for addressing these topics, which we regard as fundamental for high-altitude ecosystem establishment.

In addition to *Pseudomonas*, we found Mortierellaceae associated to bacteria: *Rahnella, Rouxiella*, and *Janthinobacterium.* While some studies showed a positive influence of *Pseudomonas*, *Rahnella*, and *Janthinobacterium* on plant growth (Kang et al., 2007; Vyas et al., 2010; Chatterjee et al., 2017), such information remain scarce for fungal–bacterial associations. Particularly, *Pseudomonas* and *Rahnella* were reported to use fungal hyphae as highways (Palmieri et al., 2020; Steffan et al., 2020), while they were not reported to occur within fungal hyphae. The question of mutualism between fungi, more specifically *Mortierella*, and epihyphal bacteria has not been fully addressed yet.

For several fungal phyla, including Glomeromycotina (arbuscular mycorrhizal fungi), Mucoromycotina, and Mortierellomycotina, bacterial endohyphal symbionts have been extensively studied so far. For Mortierellomycotina, both *Burkholderia-* and *Mycoplasma*-related endohyphal symbionts have been reported (Uehling et al., 2017; Desirò et al., 2018; Takashima et al., 2018). Here, the genera *Mycoavidus, Burkholderia*, and *Paraburkholderia* were detected. The epi- or endohyphal location of the bacteria has not been within the aim of this study, which solely focused on distribution patterns. Interestingly, despite the high number of strains included in this study, *Mycoplasma-*related bacteria were not detected. This seems to be in agreement with Takashima et al. (2018) who also only reported *Burkholderia-*related bacteria in their study screening >200 Mortierellaceae strains isolated from cold, temperate and subtropical soils. Possibly, geography and environmental conditions are important factors shaping fungus–bacterial associations. Moreover, also isolation conditions might have played a role as both studies used _LC_A and our study in addition PDA medium for isolation. According to Takashima et al. (2018), however, the percentage of Mortierellaceae harboring *Burkholderia-*related endobacteria was 22%. Here, the fraction of such associations was 13%. Different habitats offer a possible explanation for the different percentages. The different species compositions among habitats, might be a further explanation, as the overlap of species considered in these studies is small. Here, higher altitude was positively associated with the frequency of associations. Taking together this information and owing to the hypothesis of species-specificity, different Mortierellaceae genera and species might associate with *Burkholderia-*related bacteria, depending on environmental conditions, which are also likely to play a role in shaping these associations.

Independent comparative phylogenomic analyses of fungal and bacterial genomes are consistent with an ancient origin for *L. elongata* – *Mycoavidus cysteinexigens* symbiosis, most likely originating over 350 million years ago and concomitant with the colonization of terrestrial habitats on Earth, and the diversification of plants and fungi (Uehling et al., 2017). If endohyphal associations have an ancient origin, the ability to form associations with bacteria could be a common synapomorphic trait in the Mortierellaceae lineage. We detected at least four bacterial species, which, based on their taxonomic placement, are possibly endohyphal (Ohshima et al., 2016; Uehling et al., 2019; Okrasiñska et al., 2021). *Mycoavidus cysteinexigens* was the most frequently detected one. This is the first time that *M. cysteinexigens* associations have been shown for alpine habitats (alpine bare terrain and *P. cembra* forests). Based on our results, *M. cysteinexigens* can be associated with a wide range of Mortierellaceae species, such as *P. horticola/verticillata*, *L. gamsii*, and *L. exigua*, all falling into the *Linnemannia/Podila* lineage (Figure 2). Endobacteria depend on the carbon and nitrogen supply by the Mortierellaceae host (Uehling et al., 2017; Desirò et al., 2018). Harboring endobacteria would come with fitness costs based on metabolomics and proteomics data (Li et al., 2017). As a fair trade, the endosymbiont would provide certain metabolites (e.g., amino acids) and chemical weapons to the host, repaying the fungi for sustaining and transporting them (Lackner et al., 2011; Steffan et al., 2020). However, it is still unclear how recognition and partner acquisition work, and whether or not bacterial endosymbionts can be horizontally transmitted. A possible reason for these endobacteria to associate with Mortierellaceae could be the ability of these fungi to grow under oligotrophic, or cold environmental conditions. Moreover, they are very frequent and are one of the dominant soil fungal groups on a global scale irrespective of the habitats.

In summary, our study provides evidence in favor of a selective facultative association hypothesis, which is established based on the presence of a suitable partner, and environmental conditions. Fungi belonging to the phylum Mortierellomycotina do not only occasionally associate with bacteria, but can also establish a wide range of associations with different bacteria, especially with selected *Pseudomonas* OTUs.

## Conclusion

Mortierellaceae species are diverse in alpine and high-altitude habitats. Their diversity depended on environmental factors, namely habitat and snow-cover, as well as environmental conditions, such as soil pH. We frequently observed associations between Mortierellaceae isolates and gram-negative bacteria belonging to seven different genera. The associations we observed were partially specific for Mortierellaceae species and genera and also driven by environmental factors, including habitat, altitude, and season. Especially the effect of altitude might indicate that these interactions are ecologically meaningful and need to be investigated further.

## Data Availability Statement

The datasets presented in this study can be found in online repositories. The names of the repository/repositories and accession number(s) can be found in the article/Supplementary Material.

## Author Contributions

AT, MP, and UP conceived the study design. AT isolated and characterized all *Mortierella* strains and was responsible for the wet lab experiments. MP contributed to the data analysis and data interpretation. AT, MP, and UP are responsible for manuscript writing and editing. UP contribute to the funding acquisition. All authors have read and agreed to the published version of the manuscript.

## Conflict of Interest

The authors declare that the research was conducted in the absence of any commercial or financial relationships that could be construed as a potential conflict of interest.
