# Supplementary material for: Habitat, Snow-Cover and Soil pH, Affect the Distribution and Diversity of Mortierellaceae Species and Their Associations to Bacteria
Source: Front Microbiol. 2021 Jul 1;12:669784. doi: 10.3389/fmicb.2021.669784 (PMC8283828; doi:10.3389/fmicb.2021.669784)
Supplement: Supplementary file 3 [file Table_3.docx]

**Supplementary Material**

**Habitat, snow-cover and soil pH, affect the distribution and diversity of Mortierellaceae species and their associations to bacteria**

Anusha Telagathoti, Maraike Probst, Ursula Peintner^*^


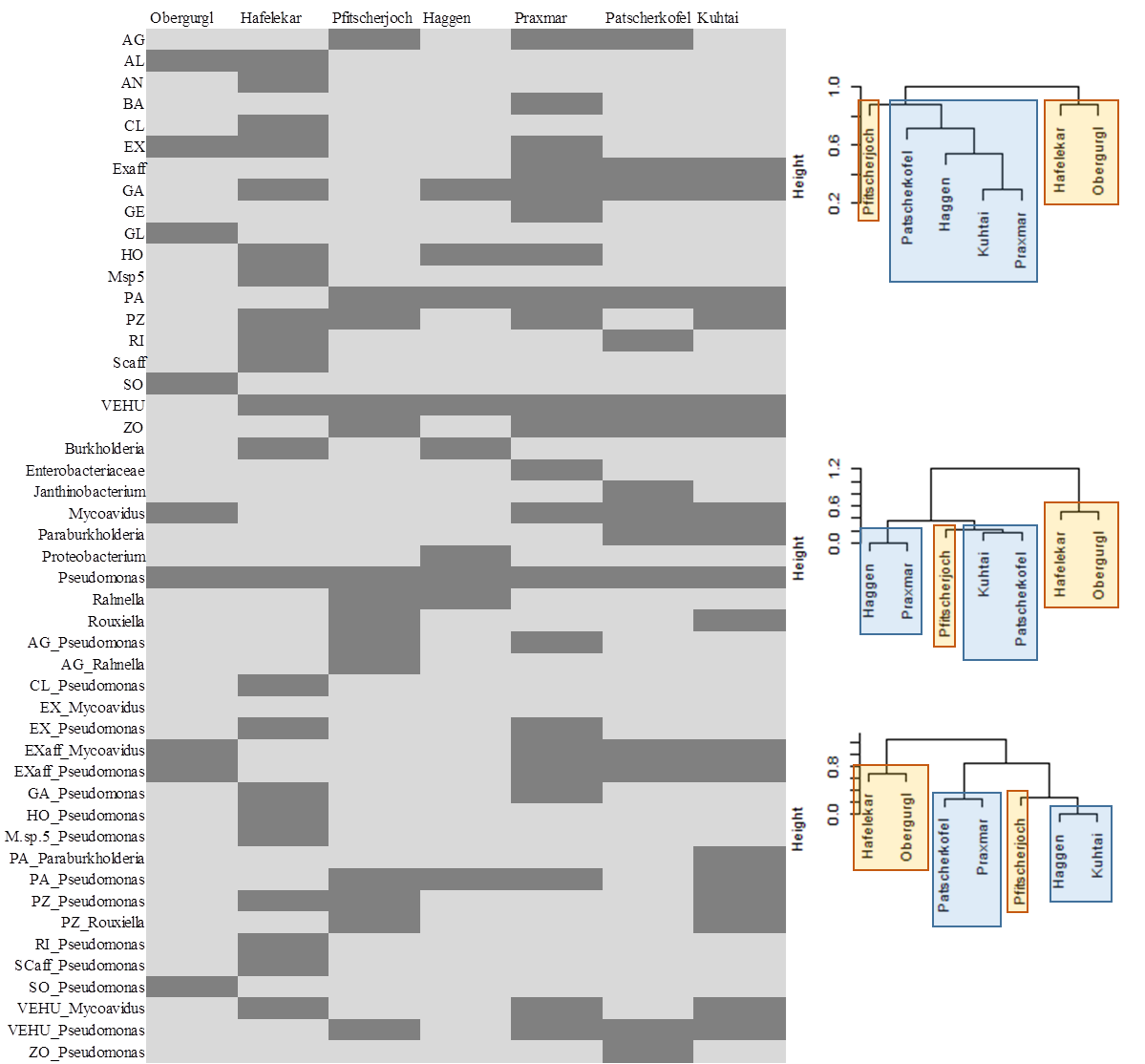


**SI Figure 1 Distribution of Mortierellaceae, associated bacteria and Mortierellaceae*-*bacteria pairs across locations.** The heatmap indicates occurrences (presence (dark grey)-absence (light grey)). Cluster dendrograms show groupings of locations.


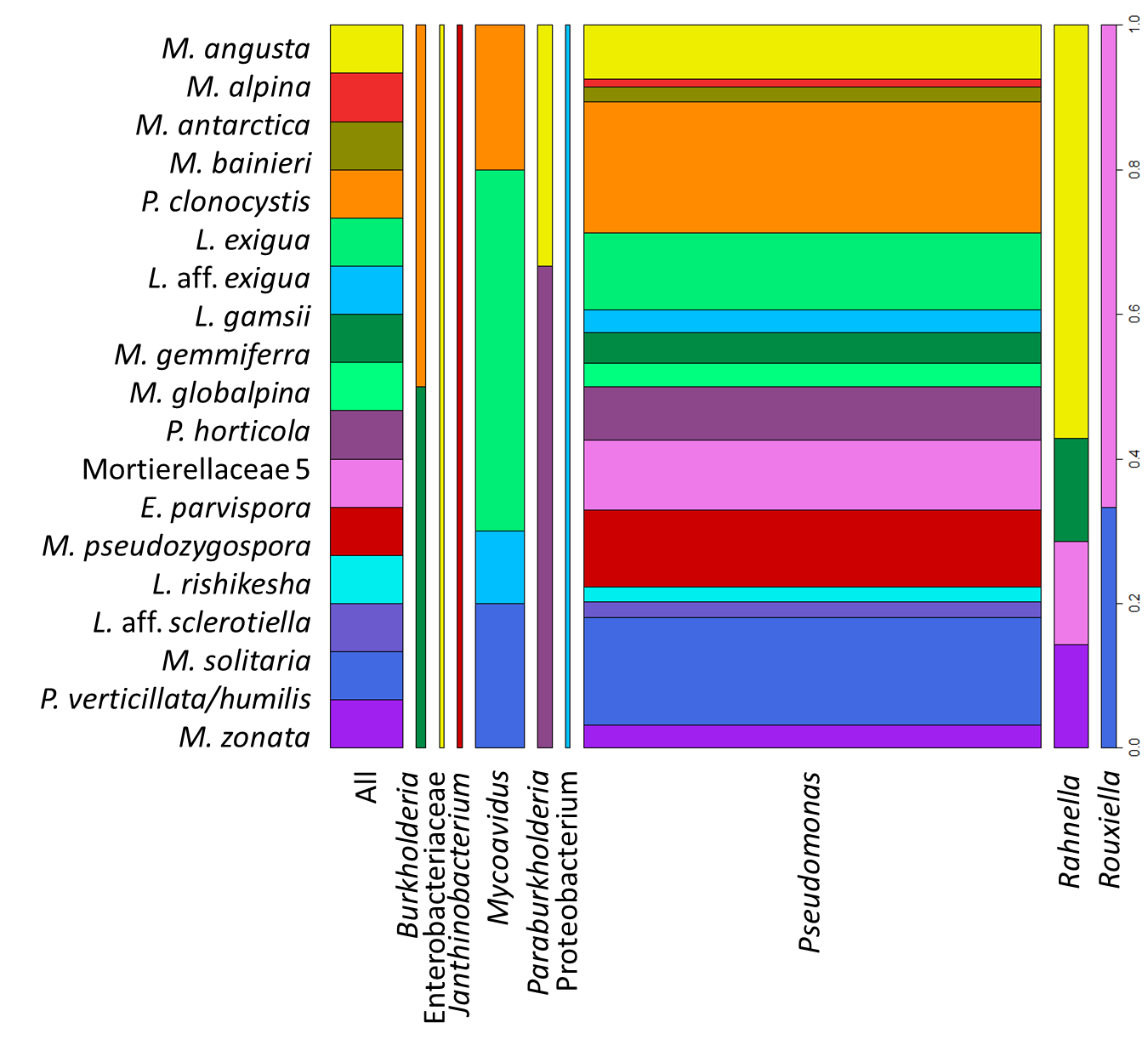


**SI Figure 2 Mortierellaceae-bacteria pairs detected in the overall dataset.** Bar width is relative to the total number of isolates. E = *Entomortierella,* L = *Linnemannia,* M = *Mortierella* (*sensu strictu: M. alpina, M. antarctica, M. globalpina;* all other *Mortierella* species need to be considered *sensu lato*)*,* P = *Podila.*


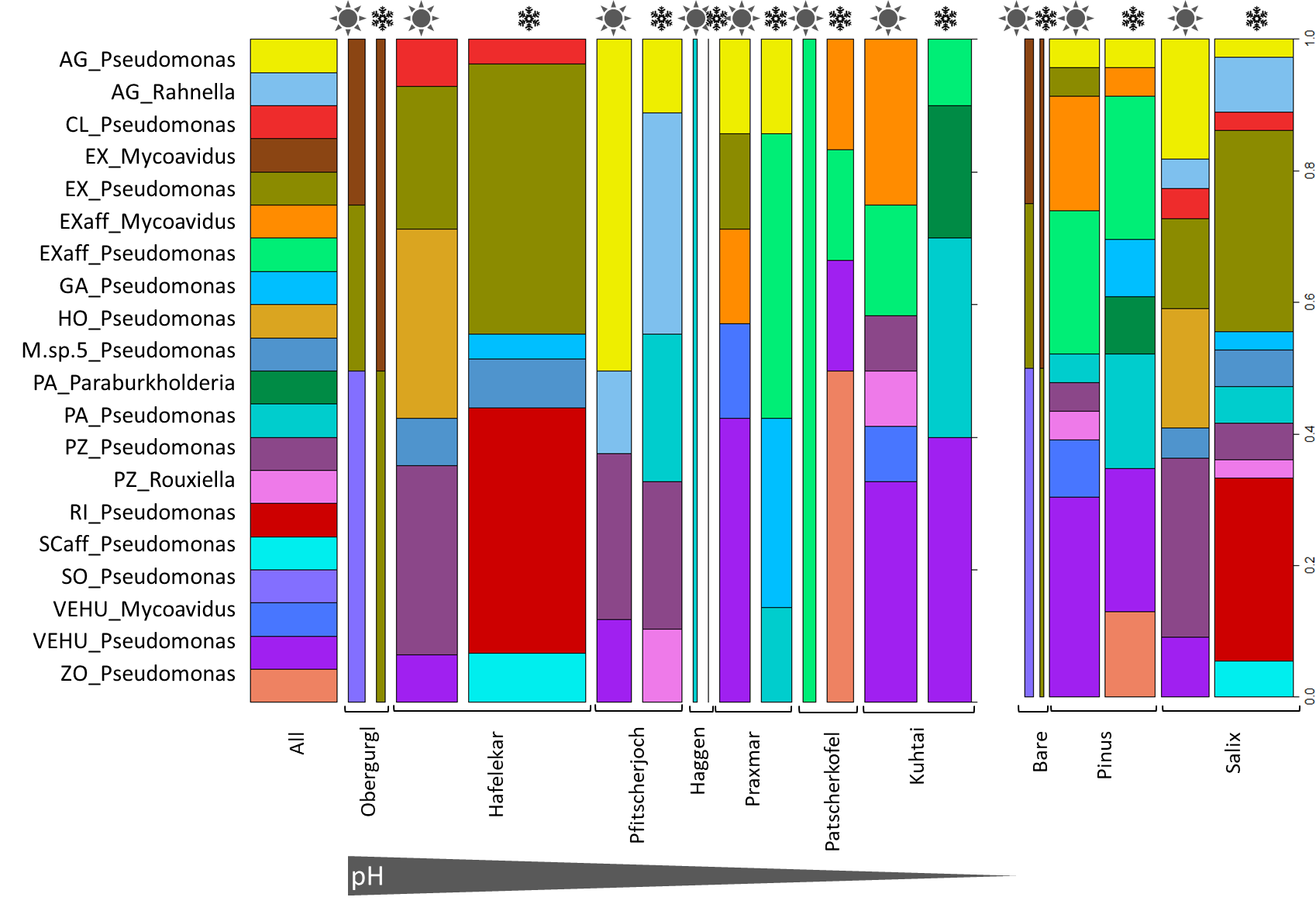


**SI Figure 3** **Distribution of Mortierellaceae*-*bacteria pairs across sampling sites (left) and habitats (right).** Stars and snowflakes indicate whether the sample, from which the pair was isolated, was covered with snow or not. Species represented by one single species were excluded from the illustration. Bar width is proportionate to the sample size of the sample group in the overall data set. AG = *M. angusta* (*s.l.*), AL = *M. alpina* (*s.s.*), AN = *M. antarctica* (*s.s.*), BA = *M. bainieri* (*s.l.*), CL = *Podila clonocystis*, Ex = *Linnemannia exigua*, Exaff = *L.* aff. *exigua*, GA = *L. gamsii*, GL = *M. globalpina* (*s.s.*), HO = *P. horticola*, M. sp. 5 = *Mortierellaceae* species complex 5, PA = *Entomortierella parvispora*, PZ = *M. pseudozygospora* (*s.l.*), RI = *L. rishikesha*, SCaff = *L.* aff. *sclerotiella*, SO = *M. solitaria* (*s.l.*), VEHU= *P. verticillata/humilis*, ZO = *M. zonata* (*s.l.*).


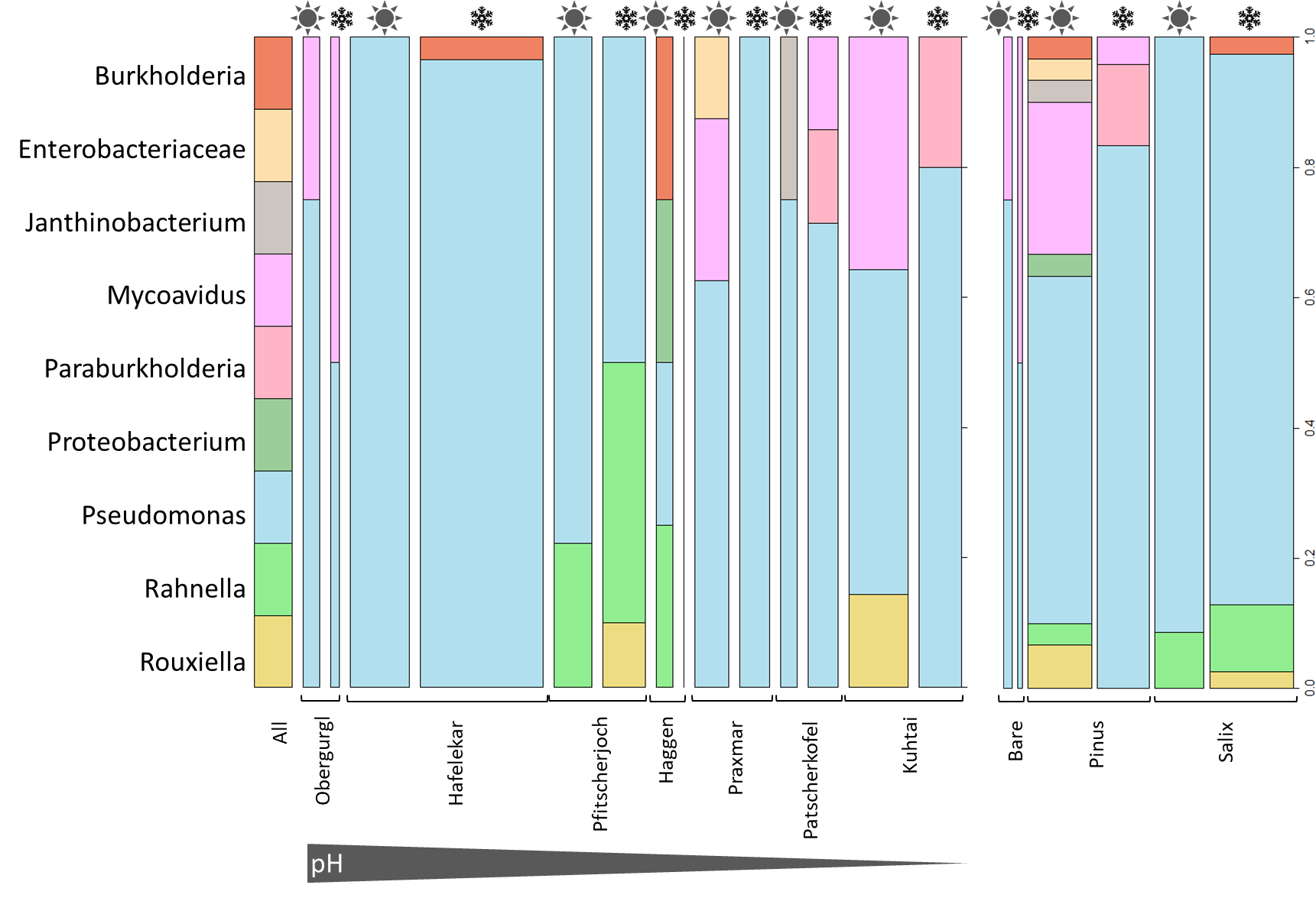


**SI Figure 4** **Distribution of bacteria associated to isolated Mortierellaceae species across sampling sites (left) and habitats (right).** Stars and snowflakes indicate whether the sample, from which the Mortierellaceae strain was isolated, was covered with snow or not, respectively. Singular events were excluded from the illustration. Bar width is proportionate to the sample size of the sample group in the overall data set.


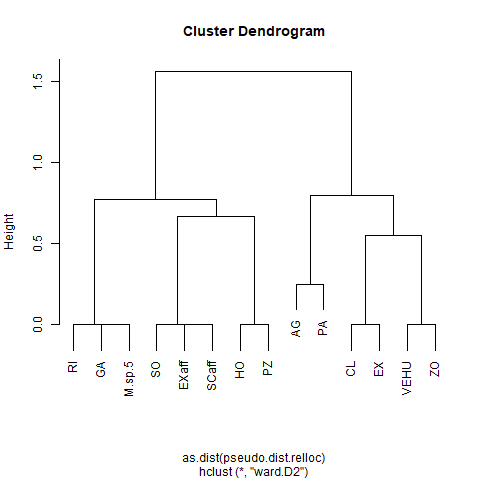


**SI Figure 5 Clustering of Mortierellaceae species by the phylogeny of their associated *Pseudomonas* bacterium.** The dataset was reduced to only Mortierellaceae isolates, in which *Pseudomonas* was detected*.* Pseudomonas isolates were grouped into OTUs based on their 16S rDNA gene sequence (according to Figure 4). The distribution of *Pseudomonas* OTUs was used in a presence-absence manner for clustering Mortierellaceae species. AG = *M. angusta* (*s.l.*), CL = *Podila clonocystis*, Ex = *Linnemannia exigua*, Exaff = *L.* aff. *exigua*, GA = *L. gamsii*, HO = *P. horticola*, M. sp. 5 = *Mortierellaceae* species complex 5, PA = *Entomortierella parvispora*, PZ = *M. pseudozygospora* (*s.l.*), RI = *L. rishikesha*, SCaff = *L.* aff. *sclerotiella*, SO = *M. solitaria* (*s.l.*), VEHU= *P. verticillata/humilis*, ZO = *M. zonata* (*s.l.*).
